# Genotype-Phenotype Associations of the CD-Associated Single Nucleotide Polymorphism within the Gene Locus Encoding Protein Tyrosine Phosphatase Non-Receptor Type 22 in Patients of the Swiss IBD Cohort

**DOI:** 10.1371/journal.pone.0160215

**Published:** 2016-07-28

**Authors:** Marianne R. Spalinger, Jonas Zeitz, Luc Biedermann, Jean-Benoit Rossel, Michael C. Sulz, Pascal Frei, Sylvie Scharl, Stephan R. Vavricka, Michael Fried, Gerhard Rogler, Michael Scharl, Swiss IBD Cohort Study Group

**Affiliations:** 1 Division of Gastroenterology and Hepatology, University Hospital Zurich, University of Zurich, Zurich, Switzerland; 2 Institute of Social and Preventive Medicine, Université de Lausanne, Lausanne, Switzerland; 3 Division of Gastroenterology and Hepatology, Kantonsspital St. Gallen, St. Gallen, Switzerland; 4 Zurich Center for Integrative Human Physiology, University of Zurich, Zurich, Switzerland; Cincinnati Children's Hospital Medical Center, UNITED STATES

## Abstract

**Background:**

Protein tyrosine phosphatase non-receptor type 22 (PTPN22) plays an important role in immune cell function and intestinal homeostasis. The single nucleotide polymorphism (SNP) rs2476601 within the *PTPN22* gene locus results in aberrant function of PTPN22 protein and protects from Crohn’s disease (CD). Here, we investigated associations of PTPN22 SNP rs2476601 in inflammatory bowel disease (IBD) patients in the Swiss IBD Cohort Study (SIBDCS).

**Methods:**

2’028 SIBDCS patients (1173 CD and 855 ulcerative colitis (UC) patients) were included. The clinical characteristics were analysed for an association with the presence of the PTPN22 SNP rs2476601 genotypes ‘homozygous variant’ (AA), ‘heterozygous’ (GA) and ‘homozygous wild-type’ (GG).

**Results:**

13 patients (0.6%) were homozygous variant (AA) for the PTPN22 polymorphism, 269 (13.3%) heterozygous variant (GA) and 1’746 (86.1%) homozygous wild-type (GG). In CD, AA and GA genotypes were associated with less use of steroids and antibiotics, and reduced prevalence of vitamin D and calcium deficiency. In UC the AA and GA genotype was associated with increased use of azathioprine and anti-TNF antibodies, but significantly less patients with the *PTPN22* variant featured malabsorption syndrome (p = 0.026).

**Conclusion:**

Our study for the first time addressed how presence of SNP rs2476601 within the PTPN22 gene affects clinical characteristics in IBD-patients. Several factors that correlate with more severe disease were found to be less common in CD patients carrying the A-allele, pointing towards a protective role for this variant in affected CD patients. In UC patients however, we found the opposite trend, suggesting a disease-promoting effect of the A-allele.

## Introduction

A single nucleotide polymorphism (SNP) within the gene locus encoding protein tyrosine phosphatase non-receptor type 22 (PTPN22; SNP ID rs2476601) has been associated with an increased risk to develop autoimmune disorders, including rheumatoid arthritis (RA)[1–3], systemic lupus erythematosus (SLE)[4–6], Graves disease[7], and type-I diabetes (T1D)[7, 8]. Interestingly, genome-wide association studies (GWAS) that addressed genes associated with inflammatory bowel disease (IBD), revealed that the very same SNP reduces the risk to develop Crohn’s disease (CD)[9–12]. While there was no association found with ulcerative colitis (UC) in most of these studies, one of them found a moderate decrease in UC disease risk, which was attributed to correlation with reduced TNF serum levels[9]. In contrast to classical autoimmune or auto-inflammatory disorders, where the adaptive immune system attacks the body’s own cells/tissues, current hypothesis suggest that IBD is driven by inflammatory reactions against the harmless commensal microbiota in the intestine[13–15]. It has been suggested that genetic factors result in a defective innate immune response towards invading intestinal pathogens ultimately driving an over-activation of the adaptive arm of the immune system, what finally causes severe chronic and/or relapsing intestinal inflammation[13, 14, 16–18]. Although up to date over 200 gene loci have been associated with an altered risk to develop IBD[19], and for several of them, basic research has provided important mechanistic insight, it is still not known how presence of these SNPs affects clinical outcome and/or disease characteristics in IBD patients.

The CD-associated SNP rs2476601 is located in exon 14 of the *PTPN22* gene locus and results in the substitution of arginine 620 with a tryptophan residue in the PTPN22 protein product (PTPN22-620W). Although initial studies demonstrated that presence of the variant results in increased *in vitro* dephosphorylation capacity[20], the PTPN22-620W variant is nowadays regarded to lead to an altered-function protein, since more recent studies demonstrated that mice designed to express the murine orthologue of PTPN2-620W, feature increased T cell receptor signaling and enhanced levels of autoreactive T cells, phenocopying the findings in PTPN22 deficient animals[21, 22]. Later, these changes in T cell receptor signaling were attributed to altered substrate specificity of the PTPN22-620W variant[23].

*PTPN22* is expressed in all immune cells, including B and T lymphocytes as well as myeloid immune cells such as monocytes, dendritic cells and macrophages[24], but not in non-hematopoietic cells such as intestinal epithelial cells or fibroblasts ([24] and own unpublished data). In T and B cells, PTPN22 activity attenuates antigen receptor signaling[20, 22, 25], ultimately promoting proliferation and aberrant activation of T and B cells[25–27]. The function of PTPN22 in innate immune cells is less studied, although it seems to be importantly involved in intestinal homeostasis: we have found that PTPN22 is reduced in intestinal biopsies of IBD patients when compared to healthy subjects[28]. This reduction was mainly due to decreased expression of PTPN22 in CD68+ cells of the monocyte/macrophage linage, while its expression in B and T cells remained unchanged[28]. Loss of PTPN22 in monocytes results in misbalanced secretion of inflammatory cytokines in response to IFN-γ and the bacterial cell wall product muramyl dipeptide, characterized by enhanced levels of IL-6 and IL-8, but decreased IL-12 and IFN-γ [28, 29]. Further, loss of PTPN22 and presence of PTPN22-620W in macrophages favors generation of pro-inflammatory M1 macrophages[30], and attenuates toll-like receptor (TLR)4 and TLR7 signaling, resulting in decreased Type-I interferon responses[31, 32]. The importance of PTPN22 in intestinal homeostasis is further demonstrated by the fact that loss of PTPN22 results in increased dextran sodium sulfate (DSS)-induced acute colitis[30, 31].

Taken together, these data describe an important role for PTPN22 in regulating inflammatory events in the intestine, but up to date, it has not been addressed how presence of the minor (A) allele influences clinical course or disease characteristics in affected patients. Therefore, here we aimed to address how SNP rs2476601 in *PTPN22* influences clinical parameters in patients suffering from IBD. Since SNP rs2476601 is differentially associated with IBD than with classical inflammatory disorders, we believe that this can give important insight to understand why SNP rs2476601 is negatively associated with CD. Further, a better understanding of the association between IBD risk loci and the complex pathophysiology of IBD might result in better prediction of the disease course and therefore might have an important impact on treatment decisions.

Using the patient collective of the Swiss IBD Cohort Study (SIBDCS), we investigated, whether presence of the CD-associated *PTPN22* variant, rs2476601 is associated with distinctive disease characteristics in Swiss IBD patients.

## Results

### Distribution of PTPN22 alleles in the SIBDCS CD and UC patient cohorts

We analysed a total of 2’028 IBD patients from the SIBDCS, consisting of 1173 (57.8%) CD and 855 (42.2%) UC patients. Of the entire set of patients, 13 patients (0.6%) featured the AA genotype of the *PTPN22* polymorphism, 269 (13.3%) carried the heterozygous form (GA) and 1’746 (86.1%) the homozygous wild-type (GG).

In the group of 1173 CD patients 1034 (88.2%) carried the homozygous wild-type allele (GG) of PTPN22, 136 (11.6%) the heterozygous form (GA) and 3 (0.3%) the AA genotype. In the UC group 712 (83.3%) carried the homozygous wild-type allele (GG), 133 (15.6%) the heterozygous form (GA) and 10 (1.2%) the AA genotype. The groups GA and AA were merged together to compare existence of the A-allele to its non-existence. When comparing the distribution of these genotypes between the CD and UC group, according to a chi-squared test, the distributions of these genotypes were significantly different (p = 0.002; Table 1).

**Table 1:** Distribution of genotypes in UC and CD patients.

Number (%)	GG	GA or AA	p-value (chi2)
Diagnosis			
Crohn (1173 patients)	1034 (59.22%)	139 (49.29%)	**0.002**
UC or IC (855 patients)*	712 (40.78%)	143 (50.71%)	

*There are 804 UC and 51 IC patients.

### CD patients carrying the A-allele are treated less often with steroids or antibiotics

**Fig 1:**
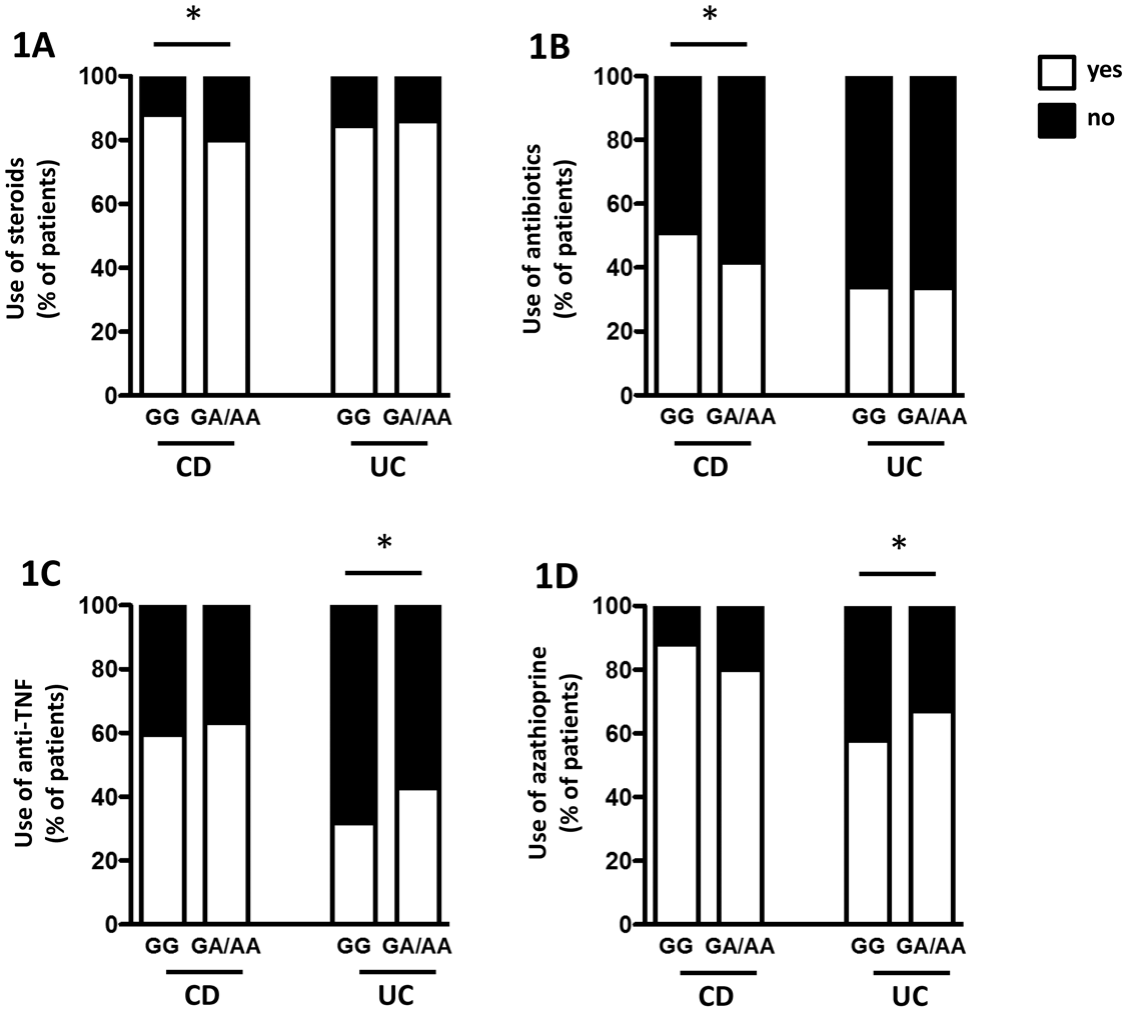
Use of steroids, antibiotics, anti-TNF treatment, and azathioprine in CD and UC patients carrying the A-allele in SNP rs2476601. The graphs show percentage of CD (left two bars in each graph) and UC patients (right two bars in each graph) treated (white area) or not treated (black area) with **A:** steroids, **B:** antibiotics, **C:** anti-TNF medication, **D:** or azathioprine.

Disease course and response to a certain treatment are crucial clinical parameters to determine further treatment options, and they might be a factor to evaluate overall disease severity (e.g. anti-TNF antibodies are frequently used in patients refractory to other treatment approaches). Therefore, we next addressed whether presence of the A-allele was associated with specific medications and/or response to medication. In the CD group, the distribution of genotypes significantly differed between patients with steroid therapy (910 GG / 111 GA or AA) and those without steroid therapy (124 GG / 28 GA or AA), which was verified using the chi-squared test (p-value: 0.007; Fig 1A, S1 Table). This was also statistically significant when comparing the number of follow-ups with a therapy using steroids (p-value 0.034 by a chi-squared test; S1 Table). Also for the use of antibiotics the distribution of genotypes for those patients with antibiotic therapy (525 GG / 58 GA or AA) significantly differed from the CD patients not using antibiotics (509 GG / 81 GA or AA) with a p-value of 0.045 (Fig 1B: S1 Table). When analysing the use of anti-TNF antibodies, failure or non-response to anti-TNF therapy, non-response to steroids, use of azathioprine and/or 6-mercaptopurine, as well as use of methotrexate, cyclosporine and/or tacrolimus, no significant difference could be detected between the genotypes (GG versus GA/AA; Fig 1C+1D and S1 Table).

### In UC patients use of anti-TNF antibodies and use of steroids is enhanced with the AA or GA genotype

In the UC group, the distribution of genotypes for patients with use of anti-TNF therapy (227 GG / 61 GA or AA) significantly differed from the UC patients not using anti-TNF therapy (485 GG / 82 GA or AA) with a p-value of 0.013 by a chi-squared test (Fig 1C). The allele distribution in UC patients using azathioprine, but not in those using 6-mercaptopurine, also differed significantly (411 GG / 96 GA or AA versus 301 GG / 47 GA or AA; p-value 0.037; Fig 1D). This stayed significant when combining the use of azathioprine/6-mercaptopurine (437 GG / 100 GA or AA with AZA/6-MP versus 275 GG / 43 GA or AA without; p-value 0.049; S2 Table). When analysing the failure or non-response to anti-TNF therapy, use of steroids, number of follow-ups with a therapy of steroids, non-response to steroids, use of antibiotics, as well as use of methotrexate, cyclosporine and/or tacrolimus showed no significant difference when comparing genotypes (GG versus GA/AA; Fig 1 and S2 Table).

### Presence of the A-allele is not associated with markers predicting complicated disease course

**Fig 2:**
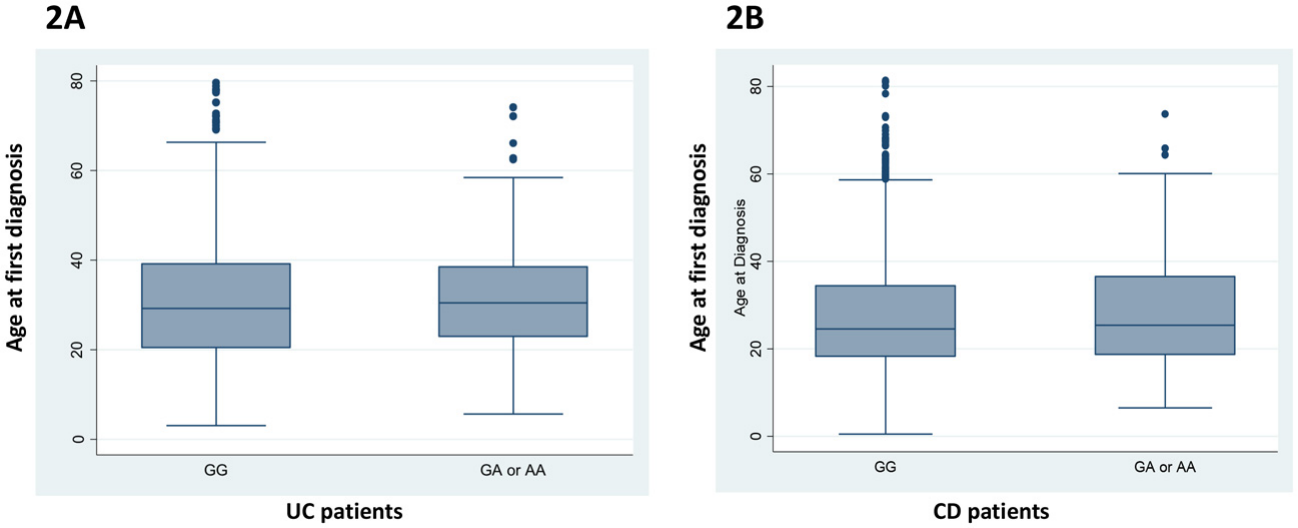
No difference in age at diagnosis in UC and CD patients carrying the A-allele in SNP rs2476601. The graphs show median age at first diagnosis (bold horizontal line), values within the 25% and 75% percentile (Box borders), and outliers (dots) in **(A)** UC, and **(B)** CD patients homozygous for the mayor (G) allele or heterozygous/homozygous carriers of the minor (A) allele in PTPN22 SNP rs2476601.

Since factors associated with a more severe disease course (e.g. IL-10R polymorphisms[33, 34], NOD2 variants[35, 36]) may also result in an earlier disease onset, we next analysed whether the age at first diagnosis is different in patients carrying the A-allele (AA and GA genotype) from those who do not (GG-genotype). In the CD group, median age at diagnosis was 24.6 years (q25-q75: 18.3–34.5; min-max: 0.5–81.4) in the GG group, and 25.4 years (q25-q75: 18.7–36.6; min-max: 6.5–73.7) in the GA or AA group, hence there was no statistically difference detectable (p = 0.28). Also in the UC group there was no significant difference in the age at diagnosis between the genotypes (GG versus GA/AA) with a median age at diagnosis of 29.0 years (q25-q75: 20.4–39.2; min-max: 3.1–79.6) within the GG genotype and 30.3 years (q25-q75: 23.0–38.5; min-max: 5.7–74.1) within the GA or AA genotype (p = 0.53; S3 Table; Fig 2A+2B). Next, we addressed demographic parameters and clinical phenotypes including gender, initial or current disease location, history of surgery, history of stenosis or fistulae and extra-intestinal manifestations in CD patients, but no significant differences between the genotypes were detected when using a chi-squared test (Table 2).

**Table 2:** Association of PTPN22 rs2476601 SNP with clinical parameters in CD.

Number (%)	GG	GA or AA	p-value (chi2)
**Gender distribution**			
**Men**	**524 (50.68%)**	**69 (49.64%)**	0.818
**Women**	510 (49.32%)	70 (50.36%)	
**Disease location**			
**Initial disease location**			
**L1**	203 (21.50%)	29 (22.66%)	
**L2**	209 (22.14%)	27 (21.09%)	
**L3**	523 (55.40%)	69 (53.91%)	0.548
**L4**	9 (0.95%)	3 (2.34%)	
***Unknown***	90 missing	11 missing	
**Current disease location**			
**L1**	221 (23.14%)	33 (25.78%)	
**L2**	211 (22.09%)	28 (21.88%)	
**L3**	514 (53.82%)	64 (50.00%)	0.449
**L4**	9 (0.94%)	3 (2.34%)	
***Unknown***	79 missing	11 missing	
**Fistula, stenosis, surgery**			
**Perianal fistula**			
**No**	778(75.24%)	102 (73.38%)	0.634
**Yes**	256 (24.76%)	37 (26.62%)	
**Other fistula**			
**No**	816 (78.92%)	119 (85.61%)	0.065
**Yes**	218 (21.08%)	20 (14.39%)	
**Anal fissure**			
**No**	896 (86.65%)	126 (90.65%)	0.187
**Yes**	138 (13.35%)	13 (9.35%)	
**Abscess**			
**No**	753 (72.82%)	98 (70.50%)	0.565
**Yes**	281 (27.18%)	41 (29.50%)	
**Summary of History of fistula**			
**No**	525 (50.77%)	72 (51.80%)	0.820
**Yes**	509 (49.23%)	67 (48.20%)	
**Stenosis**			
**No**	576 (55.71%)	75 (53.96%)	0.697
**Yes**	458 (44.29%)	64 (46.04%)	
**Surgery**			
**No**	498 (48.16%)	66 (47.48%)	0.956
**Yes**	536 (51.84%)	73 (52.52%)	
**Psoriasis and TBC**			
**Psoriasis**			
**No**	923 (89.26%)	18 (12.7)	0.836
**Yes**	111 (10.74%)	124 (87.3)	
**TBC infection**			
**No**	924 (89.36%)	18 (12.7)	0.810
**Yes**	110 (10.64%)	124 (87.3)	
**Summary of Psoriasis and TBC infection**			
**No**	925 (89.46%)	18 (12.7)	0.784
**Yes**	109 (10.54%)	124 (87.3)	
**Complications**			
**Osteopenia (–porosis)**			
**No**	777 (75.15%)	114 (82.01%)	0.075
**Yes**	257 (24.85%)	25 (17.99%)	
**Deep Venous Thrombosis**			
**No**	1010 (97.68%)	135 (97.12%)	0.686
**Yes**	24 (2.32%)	4 (2.88%)	
**Pulmonary Embolism**			
**No**	1019 (98.55%)	138 (99.28%)	0.485
**Yes**	15 (1.45%)	1 (0.72%)	
**Gallstone**			
**No**	973 (94.10%)	132 (94.96%)	0.683
**Yes**	61 (5.90%)	7 (5.04%)	
**Nephrolithiasis**			
**No**	981 (94.87%)	136 (97.84%)	0.123
**Yes**	53 (5.13%)	3 (2.16%)	
**Malabsorption syndrome**			
**No**	956 (92.46%)	130 (93.53%)	0.652
**Yes**	78 (7.54%)	9 (6.47%)	
**Perforation / Peritonitis**			
**No**	989 (95.65%)	131 (94.24%)	0.455
**Yes**	45 (4.35%)	8 (5.76%)	
**Adv. Effect of Treatment**			
**No**	899 (86.94%)	122 (87.77%)	0.785
**Yes**	135 (13.06%)	17 (12.23%)	
**Summary of Complications**			
**No**	590 (57.06%)	82 (58.99%)	0.665
**Yes**	444 (42.94%)	57 (41.01%)	
**Extraintestinal manifestations**			
**Peripheral arthritis**			
**No**	514 (49.71%)	71 (51.08%)	0.908
**Yes**	520 (50.29%)	68 (48.92%)	
**Uveitis / Iritis**			
**No**	900 (87.04%)	125 (89.93%)	0.314
**Yes**	134 (12.96%)	14 (10.07%)	
**Pyoderma gangrenosum**			
**No**	1019 (98.55%)	137 (98.56%)	0.987
**Yes**	15 (1.45%)	2 (1.44%)	
**Erythema nodosum**			
**No**	958 (92.65%)	127 (91.37%)	0.600
**Yes**	76 (7.35%)	12 (8.63%)	
**Aphtous oral ulcers**			
**No**	877 (84.82%)	124 (89.21%)	0.245
**Yes**	157 (15.18%)	15 (10.79%)	
**Ankylosing spondylitis**			
**No**	942 (91.10%)	127 (91.37%)	0.871
**Yes**	92 (8.90%)	12 (8.63%)	
**Prim. scler. cholangitis**			
**No**	1026 (99.23%)	136 (97.84%)	0.113
**Yes**	8 (0.77%)	3 (2.16%)	
**Other**			
**No**	974 (94.20%)	133 (95.68%)	0.469
**Yes**	60 (5.80%)	6 (4.32%)	
**Summary of Extraintestinal manifestations**			
**No**	412 (39.85%)	58 (41.73%)	0.793
**Yes**	622 (60.15%)	81 (58.27%)	

### Association with malabsorption in UC patients and vitamin D and calcium deficiency in CD patients

**Fig 3:**
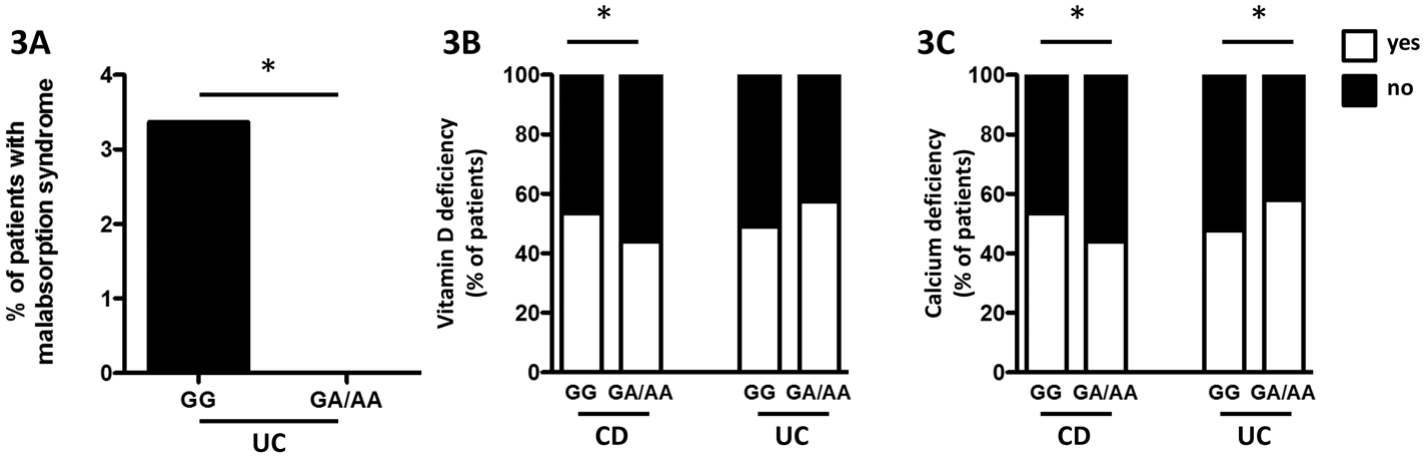
Presence of SNP rs2476601 affects malabsorption, vitamin D and calcium deficiency. **A:** percentage of UC patients with malabsorption syndrome. **B+C:** percentage of CD (left two bars) and UC patients (right two bars) featuring (white area) or not featuring (black area) **B:** vitamin D deficiency, or **C:** calcium deficiency.

We next analysed, whether PTPN22 variation might be associated with malabsorption and vitamin deficiency in IBD patients. In the UC group, malabsorption syndrome showed a statistically different distribution between the genotypes with 24 (3.37%) patients with malabsorption syndrome in the GG group compared to 0% in the GA or AA group (P = 0.026; Fig 3A and S3 Table). Malabsorption is neither well defined in the Swiss IBD cohort nor in the gastroenterological literature in general, and its occurrence in UC patients is rather uncommon. Further, deficiency of vitamin K and vitamin D are associated with active intestinal inflammation[37, 38]. Analysis of these factors revealed that CD patients carrying the A-allele suffered less often from vitamin D and calcium deficiency (Fig 3B+3C and Table 3). In UC patients, however, presence of the A-allele was associated with increased occurrence of vitamin D deficiency (Fig 3B and Table 4), while for none of the other analyzed factors the distribution was significantly different between the genotypes (Tables 3+4).

**Table 3:** Association of PTPN22 rs2476601 SNP with BMI and micronutrient deficiency in CD patients.

Crohn’s disease patients	GG	GA or AA	p-value (Wilcoxon)
**BMI**			
Median (q25 –q75, Min–Max)	22.7 (20.2–25.8, 12.5–48.1)	22.3 (19.5–25.9, 15.4–37.0)	0.424
Unknown: 11	*9 missing*	*2 missing*	
**Hb level**			
Median (q25 –q75, Min–Max)	136.7 (128.2–145.8, 83–179)	138.2 (129–147, 97.5–168)	0.428
Unknown: 6	*4 missing*	*2 missing*	
**Vit. B12 deficiency**			
No	684 (76.68%)	88 (76.52%)	
Yes	208 (23.32%)	27 (23.48%)	0.970
Unknown: 166	*142 missing*	*24 missing*	
**Folate deficiency**			
No	499 (64.14%)	64 (68.82%)	
Yes	279 (35.86%)	29 (31.18%)	0.372
Unknown: 302	*256 missing*	*46 missing*	
**Anaemia**			
No	625 (60.68%)	89 (64.96%)	
Yes	405 (39.32%)	48 (35.04%)	0.334
Unknown: 6	*4 missing*	*2 missing*	
**Iron def. Anaemia**		*17 missing*	
No	787 (84.26%)	106 (86.89%)	
Yes	147 (15.74%)	16 (13.11%)	0.451
Unknown: 117	*100 missing*		
**Iron def. + chronic disease anemia**			
No	920 (98.50%)	122 (100%)	
Yes	14 (1.50%)	0 (0%)	0.173
Unknown: 117	*100 missing*	*17 missing*	
**Calcium**			
No	523 (50.58%)	83 (59.71%)	
Yes	511 (49.42%)	56 (40.29%)	**0.043**
Unknown: 0			
**Vitamin D**			
No	480 (46.42%)	78 (56.12%)	
Yes	554 (53.58%)	61 (43.88%)	**0.032**
Unknown: 0			
**Folic acid**			
No	733 (70.89%)	101 (72.66%)	
Yes	301 (29.11%)	38 (27.34%)	0.665
Unknown: 0			

**Table 4:** Association of PTPN22 rs2476601 SNP with BMI and micronutrient deficiency in UC patients.

UC / IC patients	GG	GA or AA	p-value (Wilcoxon)
**BMI**			
Median (q25 –q75, Min–Max)	23.2 (20.9–26.2, 14.3–45.9)	22.9 (21.0–25.9, 13.6–48.8)	0.758
Unknown: 8	*6 missing*	*2 missing*	
**Hb level**			
Median (q25 –q75, Min–Max)	136 (127.5–145.7, 70–175)	137 (127.6–147.3, 86.5–168)	0.337
Unknown: 22	*20 missing*	*2 missing*	
**Vit. B12 deficiency**			
No	463 (85.42%)	84 (80.00%)	
Yes	79 (14.58%)	21 (20.00%)	0.159
Unknown: 208	*170 missing*	*38 missing*	
**Folate deficiency**			
No	328 (68.19%)	59 (62.77%)	0.305
Yes	153 (31.81%)	35 (37.23%)	
Unknown: 280	*231 missing*	*49 missing*	
**Anaemia**			
No	406 (58.67%)	87 (61.70%)	
Yes	286 (41.33%)	54 (38.30%)	0.504
Unknown: 22	*20 missing*	*2 missing*	
**Iron def. Anaemia**			
No	505 (83.33%)	108 (87.10%)	
Yes	101 (16.67%)	16 (12.90%)	0.298
Unknown: 125	*106 missing*	*19 missing*	
**Iron def. + chronic disease anemia**			
No	581 (95.87%)	116 (93.55%)	
Yes	25 (4.13%)	8 (6.45%)	0.256
Unknown: 125	*106 missing*	*19 missing*	
**Calcium**			
No	373 (52.39%)	60 (41.96%)	
Yes	339 (47.61%)	83 (58.04%)	**0.023**
Unknown: 0			
**Vitamin D**			
No	364 (51.12%)	61 (42.66%)	
Yes	348 (48.88%)	82 (57.34%)	0.065
Unknown: 0			
**Folic acid**			
No	596 (83.71%)	119 (83.22%)	
Yes	116 (16.29%)	24 (16.78%)	0.885
Unknown: 0			

## Discussion

In our study, we analyzed whether presence of SNP rs2476601 within *PTPN22* is associated with disease characteristics in patients suffering from IBD. Using the longitudinal and prospectively obtained data from the SIBDC, we found that CD patients carrying the A-allele need less often steroids and/or antibiotic treatment, while no difference was detected regarding the use of anti-TNF antibodies. CD patients with the GA or AA genotype further suffer less often from vitamin D and calcium deficiency. In UC patients, presence of the A-allele was associated with enhanced use of anti-TNF medication and reduced prevalence of malabsorption syndrome, but at the same time—and in line with more severe disease—vitamin D deficiency was more common in those patients. In our study population, however, no significant difference could be found between genotypes when analysing other markers of (severe) disease, such as gender, initial or current disease location, surgery, history of stenosis or fistula and extra-intestinal manifestations.

GWAS previously associated SNP rs2476601 with reduced risk for developing CD, since this variant is less prevalent in CD patients than in the normal population. In the here presented study, we expanded this knowledge to affected IBD patients, where we found that even in patients suffering from CD, SNP rs2476601 seems to have some protective effects: steroids are usually used in more severe disease, and the use of antibiotics typically results from complications and/or infections, hence reduced use of these two medications indicate that the existence of the A-allele might protect from (severe and/or complicated) CD or might lead to a milder/less complicated disease course. Nevertheless, mechanistic data directly supporting these findings are lacking, hence our conclusion regarding the influence on disease severity should be regarded with caution. Since vitamin D deficiency is associated with active disease and a more severe disease course, the reduced abundance of vitamin D deficiency in CD patients with the GA or AA genotype further supports our hypothesis that the A-allele might be protective in CD. From basic research, it is not obvious why presence of SNP rs2476601 would result in reduced disease severity in CD. Most studies demonstrated that presence of the A-allele results in changes in T-cell responses, ultimately promoting inflammatory T cell subsets[23, 25, 39], and during innate immune reactions, presence of the A-allele has been shown to promote inflammatory macrophages[30], also indicating an inflammation-prone phenotype. However, in contrast to other inflammatory disorders, in the intestine rapid clearance of invading pathogens is crucial for homeostasis, and it might well be that the more inflammation prone nature of the (first) immune response in A-allele carriers might result in a faster clearance of infections in an early stage of the disease, preventing the development of more severe infections needing antibiotic treatment, as well as the development of progressed chronic inflammation.

Of special interest are the findings in the UC patient group: even though in most GWAS no association of *PTPN22* SNP rs2476601 with UC was found[11, 12], and one study in a Danish IBD cohort even found reduced risk to develop UC upon presence of SNP rs2476601[9], our study in contrast suggests that the A-allele might have a disease-promoting role. In contrary to the before mentioned studies, we addressed clinical associations, i.e. how the variants influences disease course in IBD, rather than the risk to develop the disease in a first place, which might explain these opposed observations. In particular, we found that UC patients with the GA or AA genotype needed anti-TNF medication more often than patients with the GG genotype. Since anti-TNF medication is usually used in more severe, treatment refractory disease, this might be an indication that presence of the A-allele possibly results in a more pronounced disease course in UC patients. Direct mechanistic data to support this finding are lacking, but some data, describing how PTPN22 affects cellular pathways involved in IBD, have been published recently[28, 29, 40] and are reviewed elsewhere[41]. There was no difference for the use of 6-mercaptopurine (6-MP), but the use of azathioprine (AZA) was significantly enhanced in UC patients carrying the A-allele, what again indicates a promoting role for the variant. Taken together this suggests that presence of the A-allele might have relevance not only for CD but also for UC patients. However, presence of the A-allele was not associated with altered response neither to the use of anti-TNF medication, nor the use of antibiotics nor steroids. This is consistent with previous findings in the above-mentioned Danish cohort, where the A-allele was also not found to be associated with changes in the response to anti-TNF treatment [9].

It might be surprising that the same genetic variant shows opposite effects on disease severity in CD and UC. The evidence pointing towards enhanced disease severity in UC is well in line with the A-allele being associated with increased risk for other autoimmune disorders. On the other hand, it is not surprising that the A-allele has a protective effect in CD, since GWAS have associated this allele with reduced risk to develop CD.

From a mechanistic point of view, the opposite findings on CD and UC disease characteristics might be explained by the fact that the PTPN22 variant affects T cell biology[22, 25], as well as pro-inflammatory signalling in tissue macrophages [30]. It is clear that the role of T cell biology is different between UC and CD, with UC classically being regarded as a Th2-mediated disorder, while in CD Th1-signature cytokines play a dominant role[42]. Therefore, changes in T cell biology, as induced by presence of the PTPN22 variant, likely have different effects on UC and CD development.

Malabsorption was significantly less often found in UC patients carrying the A-allele. Malabsorption in IBD patients is mainly caused through the presence of severe inflammation of the ileum and subsequent insufficient nutrient absorption as well as previous intestinal resection. In UC, the small intestine is typically not affected, however, malabsorption is known to occur in patients with high numbers of bowel movements thus indicating more severe disease. Micronutrition deficiency is common in IBD patients, although less prevalent in UC patients than in CD patients[43, 44]. Since micronutrition deficiencies are associated with severe disease course[43, 45], the finding that less UC patients carrying the A-allele show malabsorption, somehow contradicts severe disease in those patients. However, UC patients carrying the A-allele showed calcium deficiency more often, and no other factor associated with malabsorption was affected. Taken together this again supports the hypothesis that UC patients carrying the A-allele might suffer from more sever disease.

CD patients with the GA or the AA genotype suffered less often from vitamin D and calcium deficiencies. This is of interest, since vitamin D deficiency is known as a risk factor for IBD, and is associated with active disease[37, 46]. Animal studies have further shown that vitamin D and vitamin D receptor (VDR) are important regulators of immune homeostasis: vitamin D reduces the proliferation of CD8+ cytotoxic T cells [47], and shifts the T helper cell balance away from (pro-inflammatory) Th1 and Th17 cells towards IL-10 producing Th2 and regulatory T cells[48, 49]. Further, vitamin D influences several pathways involved in IBD pathogenesis, such as NOD2 signalling and autophagy[48, 50]. Therefore, a positive influence on vitamin D levels upon presence of the A-allele might also contribute to a less severe disease course.

A drawback of our study might be that we only addressed one single SNP, and did not take other genetic variants in account that might be present in some of the patients. Of special interest in this regard is the fact that, aside the here addressed SNP rs2476601, another variant in the gene locus encoding PTPN22 (SNP rs33996649) has been described to affect susceptibility for IBD. This variant results in a loss of PTPN22 phosphatase function, and has been described to protect from the onset of UC[12]. Unfortunately the patients enrolled in the Swiss IBD cohort have not been genotyped for SNP rs33996649, therefore analysing phenotype changes associated with this variant was not possible.

Given the number of IBD-associated SNPs, it is likely that a significant number of patients might be carrying not only one, but several disease-associated SNPs. Since presence of several SNPs might have cumulative or even multiplying effects on clinical outcome, it would be of great interest to stratify patients carrying the PTPN22 SNP rs2476601 according to the presence of other genetic variants. However, since SNP rs2476601 is rather rare, there are not enough A-allele carriers within the Swiss IBD cohort to draw meaningful conclusions from such analysis.

A limitation of our study might be that we did not include healthy subjects, especially since the occurrence of SNP rs2476601 is rather low with only 0.6% in CD patients. However, in the healthy population, the SNP is more frequent (between 1–2%), and genetic variance is rather low in a small country such as Switzerland. The main focus of our study was to determine how presence of SNP rs2476601 affects disease characteristics in IBD patients; hence including healthy controls would not add significant value to achieve this aim. Further, it has already been described thoroughly that SNP rs2476601 is associated with IBD[9, 11, 12]. For these reasons, we refrained from including healthy controls in our study.

Despite these limiting factors, we can conclude that in summary, significantly fewer patients in our cohort with the PTPN22-620W variant (GA or AA genotype) were treated with steroids and antibiotics in CD, but more with azathioprine and anti-TNF antibodies in UC. Although no disease-promoting association of the PTPN22 SNP rs2476601 with UC was described before, we demonstrated, that significantly fewer UC patients carrying the variant developed malabsorption syndrome, but vitamin D and calcium deficiency was more common. These findings might suggest a milder disease course of CD but aggravated disease in UC in A-allele carriers. This opposite influence of the A-allele on CD and UC disease development supports the hypothesis that these two forms of IBD are distinct disease entities. Since PTPN22 is involved in immune cell regulation, our findings are in line with previous findings showing that UC and CD are distinct in their immunological signature[42]. Our findings are of interest, since presence of the A-allele in *PTPN22* SNP rs2476601 is associated with several autoimmune disorders, but, to the best of our knowledge, it is currently not known how the A-allele influences disease course or treatment characteristics in any of these disorders. Therefore, our study is the first to address a clinical relevance of SNP rs2476601, and helps to better understand its effect on disease course and treatment options in IBD patients.

## Materials and Methods

### Study Design

Patient data were obtained from the register of the nationwide SIBDCS, in which patients with IBD from all regions of Switzerland have prospectively been included since 2006[51]. The cohort study is supported by the Swiss National Science Foundation. The cohort goals and methodology are described elsewhere[51].

We included 2028 IBD patients that were enrolled in the study at time of data acquisition and had been previously genotyped for the CD-associated risk variant rs2476601 within the *PTPN22* gene locus. Genotyping was performed as part of an analysis of the whole Swiss IBD cohort for all SNPs that are currently known to be associated with IBD. Since UC and IC share several disease characteristics and indeterminate colitis is often managed the same as patients who have UC, UC and IC patients were pooled for the analysis in order to increase sample size. The *PTPN22* polymorphism rs2476601 occurs in three possible isoforms: homozygous wild-type (GG), heterozygous (GA), and homozygous variant (AA). The goal of this study was to analyze whether the presence of the GA- or AA-form is associated with clinical characteristics of IBD patients.

Clinical phenotypes of CD were classified regarding disease location, which was stratified into 1 of 4 groups according to the Montreal classification and analyzed separately for initial location and current location: ileal disease with or without disease limitation to the cecum (L1), a disease limited to the colon (L2), an ileal disease with disease of the colon beyond the cecum (L3), or disease of the upper gastrointestinal tract (L4). Patients with fistulae were classified into four groups: perianal fistula, other type fistula (non-perianal fistula), multiple fistulae (>1) and any type fistula. Presence of any intestinal stenosis was included in the analysis as positive for stenosis. Location of UC was classified according to the Montreal classification into proctitis (L1), left-sided colitis (L2), pancolitis (L3) or “location unknown”[52]. We also included history of intestinal surgery. Gender, age at diagnosis, smoking history, and presence of extraintestinal manifestations were taken into account. We further obtained data about current and prior treatment with 5-aminosalicylate, antibiotics, steroids, immunosuppressants (namely azathioprine/6-mercaptopurine), calcineurin inhibitors (tacrolimus, cyclosporine), and anti-TNF drugs (infliximab, adalimumab, and certolizumab) at enrollment or according to the term “ever treated with”. Anti-TNF non-response was defined as one of the following: (1) breakthrough / loss of response, (2) primary non-response (never effective), (3) therapy stop due to side effects / intolerance.

We further analysed whether A-allele carriers suffer from micronutrient deficiencies, such as iron, vitamin B12, vitamin D, calcium, or folate deficiency, which might all result from defective absorption due to severe inflammation. A further consequence of malabsorption would be a decreased body-mass-index and decreased Hb levels. Malnutrition and micronutrient deficiencies are common in IBD patients[43, 44], and are associated with more severe disease[43, 45] and longer hospitalization times[53].

### Statistical Analysis

Clinical data were retrieved from the data center of the Swiss IBD Cohort Study at the University of Lausanne. These data and additional data obtained from a review of the patients' files were entered into a database (Access 2000; Microsoft Switzerland Ltd Liab. Co., Wallisellen, Switzerland). The Statistical Package for the Social Sciences (version 21; SPSS, Chicago, IL) was used for the statistical analysis.

Crude differences about the association of the *PTPN22* variant in relation to fistulae, stenosis, smoking status, disease location, age at diagnosis, medications and history of intestinal resection surgery were assessed using the Pearson's [chi]2 test or the Fisher's exact test (Fisher's exact test used if strata comprised a sample size ≤5). A multiple logistic regression model was calculated to identify the associations for this gene variant. Differences about the association of the *PTPN22* variant in relation to age at diagnosis were assessed using a Wilcoxon rank-sum test. A p-value smaller than 0.05 was considered significant.

### Ethical considerations

The Swiss IBD cohort study is approved by the local ethical committees (IRB approval number: EK-1316, approved on 05.02.2007 by the Cantonal Ethics Committee of the Canton Zürich, Switzerland). Written informed consent was obtained before inclusion in the cohort.

## Supporting Information

S1 Table Association of PTPN22 rs2476601 SNP with treatment characteristics of CD. (DOCX)

S2 Table Association of PTPN22 rs2476601 SNP with treatment characteristics of UC. (DOCX)

S3 Table Association of PTPN22 rs2476601 SNP with age at diagnosis. (DOCX)
