# Supplementary material for: Genotype-Phenotype Associations of the CD-Associated Single Nucleotide Polymorphism within the Gene Locus Encoding Protein Tyrosine Phosphatase Non-Receptor Type 22 in Patients of the Swiss IBD Cohort
Source: PLoS One. 2016 Jul 28;11(7):e0160215. doi: 10.1371/journal.pone.0160215 (PMC4964985; doi:10.1371/journal.pone.0160215)
Supplement: S1 Table — (DOCX) [file pone.0160215.s001.docx]

| Number (%) | GG | GA or AA | p-value (chi2) |
| --- | --- | --- | --- |
| Use of Anti-TNF  No  Yes | 420 (40.62%)  614 (59.38%) | 51 (36.69%)  88 (63.31%) | 0. 375 |
| Failure or non-response to Anti-TNF therapy  No  Yes | 426 (74.2)  148 (25.8) | 61 (78.2)  17 (21.8) | 0.447 |
| Use of Steroids  No  Yes | 124 (11.99%)  910 (88.01%) | 28 (20.14%)  111 (79.86%) | **0.007** |
| Number of follow-ups with a therapy with steroids  0  1  2  3  4  5  6  7  8  9 | 124 (11.99%)  385 (37.23%)  193 (18.67%)  125 (12.09%)  75 (7.25%)  49 (4.74%)  39 (3.77%)  15 (1.45%)  15 (1.45%)  14 (1.35%) | 28 (20.14%)  45 (32.37%)  35 (25.18%)  11 (7.91%)  8 (5.76%)  5 (3.60%)  2 (1.44%)  4 (2.88%)  1 (0.72%)  0 (0%) | **0.034** |
| Non-response to steroids  No  Yes | 362 (40.86%)  524 (59.14%) | 40 (38.46%)  64 (61.54%) | 0. 638 |
| Use of Azathioprine  No  Yes  Use of 6-Mercaptopurine  No  Yes  Summary of both therapies  No  Yes | 229 (22.15%)  805 (77.85%)  879 (85.01%)  155 (14.99%)  194 (18.76%)  840 (81.24%) | 36 (25.90%)  103 (74.10%)  124 (89.21%)  15 (10.79%)  33 (23.74%)  106 (76.26%) | 0.321  0.187  0.163 |
| Use of Antibiotics  No  Yes | 509 (49.23%)  525 (50.77%) | 81 (58.27%)  58 (41.73%) | **0.045** |
| Use of Methotrexate  No  Yes  Use of Cyclosporine  No  Yes  Use of Tacrolimus  No  Yes  Summary of these therapies  No  Yes | 758 (73.31%)  276 (26.69%)  1015 (98.16%)  19 (1.84%)  1030 (99.61%)  4 (0.39%)  746 (72.15%)  288 (27.85%) | 102 (73.38%)  37 (26.62%)  136 (97.84%)  3 (2.16%)  137 (98.56%)  2 (1.44%)  101 (72.66%)  38 (27.34%) | 0.985  0.794  0.103  0.899 |

**S1 Table:** Association of PTPN22 rs2476601 SNP with treatment characteristics of CD
