# Supplementary material for: Genotype-Phenotype Associations of the CD-Associated Single Nucleotide Polymorphism within the Gene Locus Encoding Protein Tyrosine Phosphatase Non-Receptor Type 22 in Patients of the Swiss IBD Cohort
Source: PLoS One. 2016 Jul 28;11(7):e0160215. doi: 10.1371/journal.pone.0160215 (PMC4964985; doi:10.1371/journal.pone.0160215)
Supplement: S2 Table — (DOCX) [file pone.0160215.s002.docx]

| Number (%) | GG | GA or AA | p-value (chi2) |
| --- | --- | --- | --- |
| Use of Anti-TNF  No  Yes | 485 (68.12%)  227 (31.88%) | 82 (57.34%)  61 (42.66%) | **0.013** |
| Failure or non-response to Anti-TNF therapy  No  Yes | 36 (17.22%)  173 (82.78%) | 12 (21.43%)  44 (78.57%) | 0.468 |
| Use of Steroids  No  Yes | 110 (15.45%)  602 (84.55%) | 20 (13.99%)  123 (86.01%) | 0.656 |
| Number of follow-ups with a therapy with steroids  0  1  2  3  4  5  6  7  8  9 | 110 (15.45%)  215 (30.20%)  169 (23.74%)  87 (12.22%)  51 (7.16%)  29 (4.07%)  23 (3.23%)  14 (1.97%)  13 (1.83%)  1 (0.14%) | 20 (13.99%)  45 (31.47%)  32 (22.38%)  17 (11.89%)  9 (6.29%)  9 (6.29%)  7 (4.90%)  2 (1.40%)  2 (1.40%)  0 (0%) | 0.953 |
| Non-response to steroids  No  Yes | 243 (41.82%)  338 (58.18%) | 52 (44.07%)  66 (55.93%) | 0.653 |
| Use of Azathioprine  No  Yes  Use of 6-Mercaptopurine  No  Yes  Summary of both therapies  No  Yes | 301 (42.28%)  411 (57.72%)  621 (87.22%)  91 (12.78%)  275 (38.62%)  437 (61.38%) | 47 (32.87%)  96 (67.13%)  122 (85.31%)  21 (14.69%)  43 (30.07%)  100 (69.93%) | **0.037**  0.538  0.053 |
| Use of Antibiotics  No  Yes | 470 (66.01%)  242 (33.99%) | 95 (66.43%)  48 (33.57%) | 0.922 |
| Use of Methotrexate  No  Yes  Use of Cyclosporine  No  Yes  Use of Tacrolimus  No  Yes  Summary of these therapies  No  Yes | 622 (87.36%)  90 (12.64%)  658 (92.42%)  54 (7.58%)  671 (94.24%)  41 (5.76%)  566 (79.49%)  146 (20.51%) | 127 (88.81%)  16 (11.19%)  129 (90.21%)  14 (9.79%)  136 (95.10%)  7 (4.90%)  112 (78.32%)  31 (21.68%) | 0.631  0.374  0.682  0.752 |

**S2 Table:** Association of PTPN22 rs2476601 SNP with treatment characteristics of UC
