# Supplementary material for: Genotype-Phenotype Associations of the CD-Associated Single Nucleotide Polymorphism within the Gene Locus Encoding Protein Tyrosine Phosphatase Non-Receptor Type 22 in Patients of the Swiss IBD Cohort
Source: PLoS One. 2016 Jul 28;11(7):e0160215. doi: 10.1371/journal.pone.0160215 (PMC4964985; doi:10.1371/journal.pone.0160215)
Supplement: S3 Table — (DOCX) [file pone.0160215.s003.docx]

| Age at diagnosis | GG | GA or AA | p-value (Wilcoxon) |
| --- | --- | --- | --- |
| Crohn’s disease  Median, q25 – q75,  min – max | 24.6, 18.3 – 34.5,  0.5 – 81.4 | 25.4, 18.7 – 36.6,  6.5 – 73.7 | 0. 2760  *(no significant difference)* |
| Ulcerative colitis  Median, q25 – q75,  min – max | 29.0, 20.4 – 39.2,  3.1 – 79.6 | 30.3, 23.0 – 38.5,  5.7 – 74.1 | 0. 5292  *(no significant difference)* |

**S3 Table:** Association of PTPN22 rs2476601 SNP with age at diagnosis
